# Correction: 45S5 bioactive glass-based scaffolds coated with cellulose nanowhiskers for bone tissue engineering

**DOI:** 10.1039/d3ra90041j

**Published:** 2023-04-26

**Authors:** Wei Li, Nere Garmendia, Uxua Pérez de Larraya, Yaping Ding, Rainer Detsch, Alina Grünewald, Judith A. Roether, Dirk W. Schubert, Aldo R. Boccaccini

**Affiliations:** a Institute of Biomaterials, Department of Materials Science and Engineering, University of Erlangen-Nuremberg Cauerstrasse 6 91058 Erlangen Germany aldo.boccaccini@ww.uni-erlangen.de +49 9131 85 28602 +49 9131 85 28601; b Cemitec, Polígono Mocholí Plaza Cein 4 31110 Noain Navarra Spain; c Institute of Polymer Materials, Department of Materials Science and Engineering, University of Erlangen-Nuremberg Martensstrasse 7 91058 Erlangen Germany

## Abstract

Correction for ‘45S5 bioactive glass-based scaffolds coated with cellulose nanowhiskers for bone tissue engineering’ by Wei Li *et al.*, *RSC Adv.*, 2014, **4**, 56156–56164, https://doi.org/10.1039/C4RA07740G.

The authors regret unattributed data overlap between their *RSC Advances* article and ref. 1.

The SEM data in Fig. 1a of this article was re-used from ref. 1 without being correctly attributed and without permission from the Publisher.

The authors have now received the permission to reuse the images and the corrected caption is shown below:

**Fig. 1** SEM micrographs of (a) and (b) uncoated scaffolds, (c)–(f) cellulose nanowhiskers coated scaffolds, (g) cellulose nanowhiskers at different magnifications and (h) cellulose nanowhiskers coated scaffolds after immersion in SBF for 1 day. Reproduced in part from Wei Li *et al.*, *J. Mech. Behav. Biomed. Mater.*, 2014, **40**, 85–94 with permission from Elsevier.

The Royal Society of Chemistry apologises for these errors and any consequent inconvenience to authors and readers.

## Supplementary Material
